# Skeletal muscle myosin promotes coagulation by binding factor XI *via* its A3 domain and enhancing thrombin-induced factor XI activation

**DOI:** 10.1016/j.jbc.2022.101567

**Published:** 2022-01-07

**Authors:** Shravan Morla, Hiroshi Deguchi, Jevgenia Zilberman-Rudenko, András Gruber, Owen J.T. McCarty, Priyanka Srivastava, David Gailani, John H. Griffin

**Affiliations:** 1 Department of Molecular Medicine, The Scripps Research Institute, La Jolla, California, USA; 2 Departments of Biomedical Engineering and Medicine, School of Medicine, Oregon Health & Science University, Portland, Oregon, USA; 3 Departments of Pathology, Microbiology and Immunology, Vanderbilt University Medical Center, Nashville, Tennessee, USA; 4 Department of Medicine, University of California, San Diego, California, USA

**Keywords:** myosin, thrombin, coagulation factor, factor XI, skeletal muscle myosin, A, Apple, ALP, alkaline phosphatase, BLI, biolayer interferometry, BSA, bovine serum albumin, FXI, factor XI, mAb, monoclonal antibody, PK, prekallikrein, PolyP, polyphosphate, PS, phosphatidylserine, rFXI, recombinant FXI, SkM, skeletal muscle myosin, TF, tissue factor

## Abstract

Skeletal muscle myosin (SkM) has been shown to possess procoagulant activity; however, the mechanisms of this coagulation-enhancing activity involving plasma coagulation pathways and factors are incompletely understood. Here, we discovered direct interactions between immobilized SkM and coagulation factor XI (FXI) using biolayer interferometry (*K*_*d*_ = 0.2 nM). In contrast, we show that prekallikrein, a FXI homolog, did not bind to SkM, reflecting the specificity of SkM for FXI binding. We also found that the anti-FXI monoclonal antibody, mAb 1A6, which recognizes the Apple (A) 3 domain of FXI, potently inhibited binding of FXI to immobilized SkM, implying that SkM binds FXI A3 domain. In addition, we show that SkM enhanced FXI activation by thrombin in a concentration-dependent manner. We further used recombinant FXI chimeric proteins in which each of the four A domains of the heavy chain (designated A1 through A4) was individually replaced with the corresponding A domain from prekallikrein to investigate SkM-mediated enhancement of thrombin-induced FXI activation. These results indicated that activation of two FXI chimeras with substitutions of either the A3 domains or A4 domains was not enhanced by SkM, whereas substitution of the A2 domain did not reduce the thrombin-induced activation compared with wildtype FXI. These data strongly suggest that functional interaction sites on FXI for SkM involve the A3 and A4 domains. Thus, this study is the first to reveal and support the novel intrinsic blood coagulation pathway concept that the procoagulant mechanisms of SkM include FXI binding and enhancement of FXI activation by thrombin.

Surface-bound skeletal muscle myosin (SkM) promotes thrombus formation when exposed to fresh flowing human blood (1, 2), and clinical studies suggested that various plasma SkM isoforms and phenotypes are linked to pulmonary embolism and thrombin generation in plasma (1). Myosins are a large family of motor proteins sharing common features of ATP hydrolysis, actin binding, and the potential for kinetic energy transduction. SkM is a striated muscle myosin originally isolated from muscle cells that are found throughout the body, and low levels of SkM are in normal plasmas at 4 to 25 nmol/l (1). The plasma levels of SkM are elevated in patients with muscle damage (*e.g.*, acute trauma, postsurgery, postexercise, polymyositis, and rhabdomyolysis) (3, 4, 5, 6, 7, 8, 9, 10, 11), and such damaged tissues expose SkM to the blood, which can provide previously unknown sites for regulation of thrombin generation. Since bleeding in muscle is often observed in coagulation factor deficiency (12), hemostasis is a critical event in muscle damage. Therefore, it is reasonable that exposed SkM on damaged muscle may provide a procoagulant surface such that it contributes to hemostasis.

Currently, the procoagulant mechanisms of SkMs involving plasma coagulation pathways and factors are incompletely understood, indicating the need for further mechanistic studies. SkM and cardiac muscle myosin promote hemostasis and thrombotic events in murine model studies (13, 14), and they enhance prothrombin activation by clotting factor Xa in combination with factor V (1, 2, 15), an activity termed prothrombinase activity, which is associated with procoagulant anionic phospholipids that are linked to these two muscle myosins (16). Although SkM and cardiac myosin preparations are contaminated by a small amount of tissue factor (TF) (1, 16, 17), SkM is clearly very procoagulant in plasma coagulation assays even when available TF activity is inhibited by antibodies that inhibit expression of TF activity (16).

To advance knowledge about procoagulant mechanisms for SkM, here, we report studies leading to the discoveries that coagulation FXI is required for the prothrombotic activity of SkMs in fresh flowing human blood and that, in purified protein reaction mixtures, SkM binds FXI with high affinity *via* its Apple (A) 3 domain and enhances FXI activation by thrombin. These findings are the first to reveal and establish the novel concept that the procoagulant mechanisms of SkM include enhancement of FXI activation by thrombin.

## Results and discussion

New data from *ex vivo* studies of the coagulability of fresh flowing human blood over SkM-coated surfaces showed that an anti-FXI monoclonal antibody (mAb) (1A6) (18), but not a function-blocking anti-TF mAb, inhibited SkM-induced clot formation (Fig. S1), indicating that FXI activity is an essential contributor for the observed *ex vivo* procoagulant response of blood during its exposure to immobilized SkM independent of the TF pathway. This raises the question of whether the contribution of FXI to the procoagulant activity of SkM involves direct and/or indirect effects of SkM on FXI (2).

**Figure 1:**
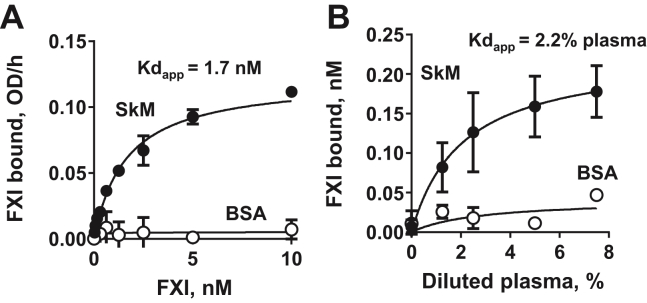
**Purified FXIand the endogenous plasma FXIbound to immobilized SkMin solid phase microtiterplate–bindingassays.** SkM or BSA was immobilized on 96-well plate in sodium bicarbonate, pH 9.3. After blocking with 0.5% BSA, aliquots (0.2 ml) containing various concentrations of purified FXI (*A*) or of diluted pooled plasma (*B*) in 30 mM Hepes buffer, pH 7.4, containing 130 mM NaCl were incubated on the immobilized SkM for 2 h at room temperature. Then, after washing the wells, the bound FXI was exposed to anti-FXI monoclonal antibody (AHXI-5061), which, after a washing step, was detected with the HRP-conjugated goat antimouse antibody as described in the Experimental procedures section. The bound endogenous plasma FXI in (*B*) was calculated using the curve shown in (*A*). Each value represents the mean (±SD) of triplicate determinations. BSA, bovine serum albumin; FXI, factor XI; HRP, horseradish peroxidase; SkM, skeletal muscle myosin.

To test the direct binding of FXI to SkM, SkM was immobilized onto microtiter plates for binding assays that employed purified FXI and endogenous FXI in plasma. In this assay system, purified FXI bound immobilized SkM (*K*_d_app = 1.7 nM) (Fig. 1*A*). The endogenous FXI in plasma also bound to immobilized SkM (*K*_*d*_app = 2.2% plasma, corresponding to approximately ∼0.7 nM FXI) (Fig. 1*B*). This implies that the exposed SkM can capture endogenous plasma FXI as well as purified FXI in model systems. Activation occurs on SkM.

**Figure 2:**
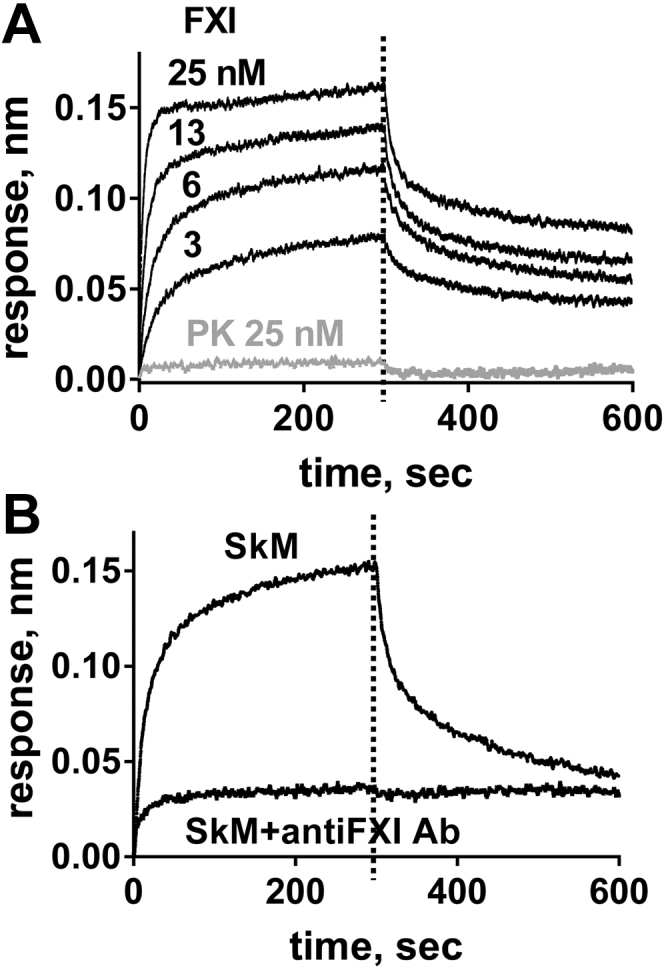
**FXI binds with high affinity to immobilized SkM.** Binding studies using biolayer interferometry (BLI) were done with FXI or prekallikrein (PK) that were in 30 mM Hepes, pH 7.4, 0.01% Tween-20 and 0.1% PEG, and 50 mM NaCl at 30 °C. SkM or BSA was immobilized onto the Octet Red Amine Reactive Second-Generation Biosensor surface. *A*, sensorgrams depict the binding of FXI or PK to immobilized SkM (concentrations from top to bottom: 25, 13, 6, 3 nM for SkM and 25 nM for PK). *B*, sensorgrams depict the binding of 12.5 nM FXI in the presence or the absence of 100 μg/ml of anti-FXI antibody (Ab), namely mAb 1A6 (18). BSA, bovine serum albumin; FXI, factor XI; SkM, skeletal muscle myosin.

To characterize in more detail, the direct interactions between SkM and FXI, biolayer interferometry (BLI) (Octet Red System) was used to record kinetics for binding of soluble FXI to immobilized SkM. BLI data showed that FXI binds to SkM with a *K*_*d*_ of 0.20 nM based on ratio of *k*_off_/*k*_on_ (*k*_on_ = 3.87 × 10^6^ M^−1^ s^−1^ and *k*_off_ = 7.95 × 10^−4^ s^−1^) (Fig. 2*A*). In contrast, 25 nM prekallikrein (PK), which is a homolog of FXI but with distinct functions, did not detectably bind to SkM under the same conditions (Fig. 2*A*), indicating the specificity of SkM for binding FXI. The anti-FXI mAb 1A6, which binds the A3 domain of FXI (18), potently inhibited binding of FXI to immobilized SkM (Fig. 2*B*), implying SkM binds to the A3 domain. Thus, BLI shows that FXI binds reversibly with a high affinity to SkM, and that the SkM-binding site is likely, at least in part, on the A3 domain of FXI.

**Figure 3:**
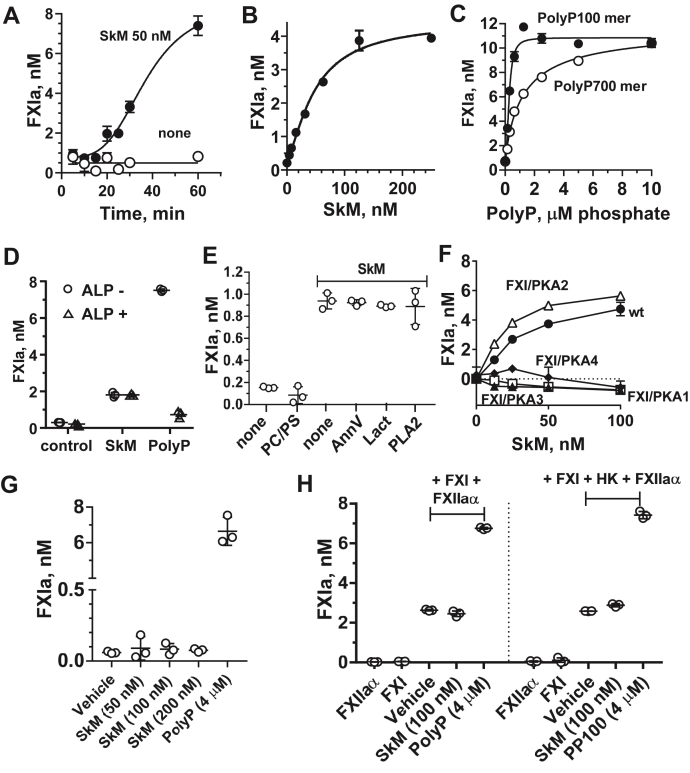
**SkM enhances FXI activation by thrombin but not FXI autoactivation or FXI activation by alpha-factor XIIa.***A*–*C*, for FXI activation by thrombin, (*A*) FXI was activated by IIa for various times in the presence of 50 nM SkM or (*B*) for 30 min at 37 °C with various concentrations of SkM or (*C*) with varying concentrations of PolyPs (100-mer [*closed circle*] or 700-mer [*open circle*]). *D* and *E*, for the effects of various reagents on SkM enhancement of FXI activation by thrombin, (*D*) FXI activation was determined in the presence of 50 nM SkM or 4 μM PolyP (100-mer) for 30 min at 37 °C and in the presence (*open triangle*) or the absence (*open circle*) of alkaline phosphatase (ALP). *E*, first, FXI activation by thrombin in the absence (“none”) or the presence of 4 μM PC/PS (80%/20% w/w) phospholipid vesicles was determined; second, FXI activation by thrombin for 30 min at 37 °C in the presence of 50 nM SkM and in the absence (“none”) or, as indicated, in the presence of annexin V (AnnV) (20 nM final), lactadherin (Lact) (20 nM, final), or phospholipase A2 (PLA2) (20 nM, final) was determined. The reactions were quenched using hirudin (2 ATU, final) to inhibit thrombin, and FXIa amidolytic activity was measured. *F*, activation of FXI/prekallikrein (PK) chimeras by thrombin: Recombinant (r) wt FXI (rFXI) (*filled circle*) or rFXI/PK Apple domain chimeras (FXI/PKA1 [*open square*], FXI/PKA2 [*open triangle*], FXI/PKA3 [*filled triangle*], or FXI/PKA4 [*filled diamond*]) were activated by thrombin in the presence of various concentrations (0–100 nM) of SkM. SkM-dependent FXIa generation after 30 min at 37 °C was determined as described in the Experimental procedures section, and FXIa levels are shown after the subtraction of the low level of FXIa generated by thrombin in the absence of SkM. *G*, FXI autoactivation and (*H*) FXI activation by alpha-factor XIIa (FXIIaα) were measured as described in the Experimental procedures section. Each value represents the mean (±SD) of triplicate determinations. FXI, factor XI; PC, phosphatidylcholine; PolyP, polyphosphate; PS, phosphatidylserine; SkM, skeletal muscle myosin.

Studies using purified clotting factors were performed to characterize select FXI-related activities that might be affected by SkM. When FXI activation by thrombin was evaluated under conditions where two differently sized polyphosphates (PolyPs), namely a ∼100-mer and a ∼700-mer, that are known to enhance FXI activation (19, 20), we found that SkM time dependently and concentration dependently enhanced FXI activation by thrombin (Fig. 3, *A* and *B*). As expected, two different PolyPs enhanced FXI activation by thrombin (Fig. 3*C*) (19, 20). Alkaline phosphatase (ALP) that hydrolyzes PolyP and ablates the ability of PolyPs to stimulate FXI activation by thrombin (19, 20) did not reduce the ability of SkMs to enhance FXI activation by thrombin (Fig. 3*D*). Thus, the activity of SkM is independent of potential PolyP contamination of SkM, and it is, in general, not susceptible to ALP. Small unilamellar phospholipid vesicles (20% phosphatidylserine [PS]/80% phosphatidylcholine [PC]) did not affect FXI activation by thrombin under the conditions employed (Fig. 3*E*). Multiple reagents that neutralize procoagulant anionic phospholipids, including lactadherin, annexin V, and phospholipase A2 (16, 17), did not affect the enhancement by SkM of FXI activation by thrombin (Fig. 3*E*). Thus, this activity of SkM is not because of anionic phospholipids linked to SkM (16). These data indicate that the SkM procoagulant activity that enhances FXI activation by thrombin is independent of PolyPs and phospholipids.

To identify protein domains in FXI involved in interactions between FXI and SkM, we employed FXI–PK chimeras because BLI-binding studies showed that, in contrast to FXI, PK did not detectably bind to SkM (Fig. 2*A*). Recombinant FXI (rFXI) proteins in which the four A domains of the FXI heavy chain (designated A1 through A4) were individually replaced with the corresponding A domain from PK were prepared. The chimeras were used in studies of FXI activation by thrombin in the presence of SkM. The FXI chimera with the substitution of the PKA1 domain (FXI/PKA1) was not significantly activated by thrombin with or without SkM, which is consistent with the known importance of the FXI A1 domain for interactions with thrombin (Fig. 3*F*) (21). Thrombin activation of the FXI chimeras with substitutions of either the A3 domains or A4 domains (FXI/PKA3 and FXI/PKA4) was not enhanced by SkM. However, substitution of the A2 domain (FXI/PKA2) did not reduce enhancement of FXI activation by thrombin compared with wt FXI (Fig. 3*F*). As noted previously (Figs. S1 and 3*B*), the anti-FXI mAb 1A6, which recognizes the A3 domain of FXIs (18), inhibited the prothrombotic activity of fresh blood flowing over a SkM-coated surface and also potently inhibited FXI binding to SkM in BLI studies. Taken together, these data strongly suggest that functional interaction site(s) on FXI for SkM involve the A3 and A4 domains of FXI. It is known that the A3 domain is needed for the procoagulant activity of FXIas and that the dimerization of FXI monomers is based on A4 domain interactions (21, 22). It is possible that A4 domain interactions between the two FXI monomers might be required for optimal interactions of FXI with SkM. To address these issues, further studies using appropriate proteins, including FXI monomers, may provide additional mechanistic insights.

The effects of SkM on FXI autoactivation and FXI activation by alpha-FXIIa were also evaluated. As is well known, PolyP and some other anionic reagents, for example, nucleic acid polymers, enhance not only FXI activation by thrombin but also FXI autoactivation and FXI activation by alpha-FXIIa (19, 21, 23). However, in contrast to PolyPs, SkM did not significantly affect FXI autoactivation (Fig. 3*G*) or FXI activation by alpha-FXIIa in the absence or the presence of high molecular weight kininogen (Fig. 3*H*). This further emphasizes that the enhancement by SkMs of FXI activation by thrombin is not because of any PolyP-like compounds and that enhancement of FXI activation by thrombin is a unique procoagulant property of SkM. In terms of broad implications, this indicates that the procoagulant activities of SkM, inter alia, include thrombin activation of FXI, which is a critical element of the intrinsic coagulation pathway's remarkable thrombin-positive feedback loops wherein thrombin activates FXI, FVIII, and FV.

FXI is a component of the intrinsic coagulation pathway, and thrombin-mediated feedback activation of FXI may contribute to hemostasis by amplification of thrombin generation through this pathway. Excessive thrombin generation is linked to thrombosis, whereas inadequate thrombin generation is linked to bleeding. Accordingly, tissue damage that exposes blood and FXI to procoagulant SkM may contribute either to hemostasis or to thrombosis, depending on many factors and circumstances. When damaged tissue enables FXI binding to SkM, it also enables FXI to colocalize with other coagulation factors that bind to SkM, such as factor VIII/von Willebrand factor complex, factor Xa, and the prothrombinase complex (1, 2), thereby integrating intrinsic pathway components and their actions.

Hereditary deficiency of FXI in humans causes a highly variable tendency to bleed excessively after trauma or surgery. Bleeding correlates poorly with plasma FXI antigen or activity levels (18, 21, 24, 25, 26, 27, 28). This clinical reality suggests that one or more as yet unknown factors modulate the hemostatic actions of FXIs. *In vitro* assays involving both clotting and fibrinolytic components may be more useful for predicting bleeding tendencies in FXI-deficient subjects than are routine clinical tests (27, 28). Such assays may more accurately portray the complexities of how FXI contributes to hemostasis. In the setting of trauma or surgery, one might consider SkM as a potential modulator of FXI-dependent thrombin generation with implications for hemostasis or thrombosis. A potential role for SkM related to FXI activity merits future preclinical and clinical studies.

In summary, we found that the *ex vivo* procoagulant activity of SkM requires FXI, SkM enhances FXI activation by thrombin, this requires the A3 and A4 domains on FXI, and the high-affinity binding of SkMs to FXI requires the FXI A3 domain. This study identifies a new mechanism for the procoagulant properties of SkM with clinical implications.

## Experimental procedures

### Materials

Rabbit SkM and phospholipase A2 from honeybee venom were purchased from Sigma–Aldrich. Human purified FXI, thrombin, bovine lactadherin, and anti-FXI mAb (AHXI-5061) were from Haematologic Technologies. Factor X and PK were from Enzyme Research Laboratories, Inc. Factor XIIaα was from Aniara Diagnostica LLC. Horseradish peroxidase–conjugated goat antimouse antibody was from Thermo Fisher Scientific. KPL SureBlue TMB 1-Component Microwell Peroxidase Substrate was from SeraCare Life Science, Inc. The chromogenic substrate for FXIa, PyrGlu-Pro-Arg-p-nitroanilide (S-2366), and H-D-Lys(Cbo)-Pro-Arg-pNA.2AcOH (Pefachrome PCa) was purchased from Diapharma and DSM Nutritional Products Ltd Branch Pentapharm, respectively. Recombinant human annexin V was from Biovision. Long-chain (p700) and medium-chain (p100) versions of PolyPs were obtained from the laboratory of James H. Morrissey, University of Michigan *via* Kerafast, Inc. Fatty acid–free and protease-free bovine serum albumin (BSA) was from Calbiochem. L-α-PS and L-α-PC (each from porcine brain) were from Avanti Polar Lipids. ALP (MB grade) was from Roche Diagnostics.

### SkM preparation

SkM was dialyzed against buffer containing 600 mmol/l NaCl, 50 mmol/l Tris, pH 7.4, and after dialysis, some particles causing turbidity were removed by high-speed centrifugation (21,130*g* for 1 min). Then SkM aliquots were stored at −80 °C until being used.

### rFXI/PK chimeras

rFXI (wt) (*circle*) or rFXI/PK A domain chimeras (FXI/PKA1, FXI/PKA2, FXI/PKA3, or FXI/PKA4) were produced and purified as described (18).

### Blood clot formation in fresh flowing human blood

*Ex vivo* studies used recalcified fresh human blood that was perfused over SkM-coated capillary surfaces or BSA-coated control surfaces under shear at 300 s^−1^ for 30 min followed by analysis of thrombus formation after fixation showing fibrin-containing thrombi, as described (2).

### The binding of purified FXI and plasma FXI to SkM immobilized in microtiter plates

SkM (100 μl at 10 μg/ml) in sodium bicarbonate buffer, pH 9.3, was immobilized to the microtiter plate for 1 h at room temperature, followed by blocking with 0.5% of BSA solution. Then, various concentrations of purified FXI (0–10 nM) or plasma with various dilutions were added to SkM-immobilized microtiter plate for 2 h at room temperature. Then, the bound FXI was incubated with anti-FXI mAb (AHXI-5061). The SkM-FXI–anti-FXI mAb complex was detected by the horseradish peroxidase–conjugated goat antimouse polyclonal antibodies with KPL SureBlue TMB 1-Component Microwell Peroxidase Substrate.

### BLI binding studies

Binding studies using BLI were done with purified FXI or PK that were in 30 mM Hepes, pH 7.4, 0.01% Tween-20, and 0.1% PEG, and 50 mM NaCl at 30 °C. SkM or BSA was immobilized onto the Octet Red Amine Reactive Second-Generation Biosensor surface prior to addition of FXI or PK.

### FXI activation by thrombin

FXI (30 nM, final) and thrombin (5 nM, final) were mixed with varying concentrations of SkM in 30 mM Hepes buffer, pH 7.4, containing 50 mM NaCl, 25 μM ZnCl_2_, and 0.1% BSA. The reaction was quenched at varying times using hirudin (two antithrombin units, final) to inhibit thrombin, and the amount of FXIa generated was measured using an FXIa chromogenic substrate (S-2366, Pyr-Glu-Pro-Arg-p-nitroanilide [Diapharma]).

### FXI/PK chimera activation by thrombin

FXI (30 nM, final) or rFXI or rFXI/PK chimeras and thrombin (5 nM, final) were mixed with 50 nM SkM in 30 mM Hepes buffer, pH 7.4, containing 50 mM NaCl, 25 μM ZnCl_2_, and 0.1% BSA. The amount of FXIa activity was measured as described previously.

### FXI autoactivation

FXI (60 nM, final) was incubated at 37 °C with SkM (50, 100, and 200 nM) or with PolyP (100-mer) (4 μM) in 30 mM Hepes, pH 7.4, buffer containing 50 mM NaCl, and 0.1% BSA. After 30 min, polybrene (6 μg/ml, final) was added to neutralize PolyP (100-mer), and FXIa amidolytic activity was measured.

### Phospholipid vesicles

Small unilamellar vesicles of 80% PC:20% PS (w/v) in the buffer containing 150 mmol/l NaCl, 50 mmol/l Tris, and pH 7.4 (TBS) were prepared by sonication under a flow of nitrogen using a microtip sonicator.

### FXI activation by alpha-factor XIIa

FXI (60 nM, final) was incubated with alpha-factor XIIa (5 nM) with SkM (100 nM) or PolyP (100 mer) (4 μM) in 30 mM Hepes, pH 7.4, buffer containing 50 mM NaCl, and 0.1% BSA in the presence or the absence of high–molecular weight kininogen (100 nM) as indicated. After 30 min at 37 °C, corn trypsin inhibitor (500 nM, final) was added to neutralize FXIIa enzyme activity, and then FXIa amidolytic activity was measured using a chromogenic substrate.

## Data availability

All data are contained within the article and supporting information.

## Supporting information

This article contains supporting information (1, 15).

## Conflict of interest

The authors declare that they have no conflicts of interest with the contents of this article.
