# Supplementary material for: Skeletal muscle myosin promotes coagulation by binding factor XI via its A3 domain and enhancing thrombin-induced factor XI activation
Source: J Biol Chem. 2022 Jan 7;298(2):101567. doi: 10.1016/j.jbc.2022.101567 (PMC8856988; doi:10.1016/j.jbc.2022.101567)
Supplement: Supplemental Figure S1 [file mmc1.pdf]

Supporting information

Figure S1

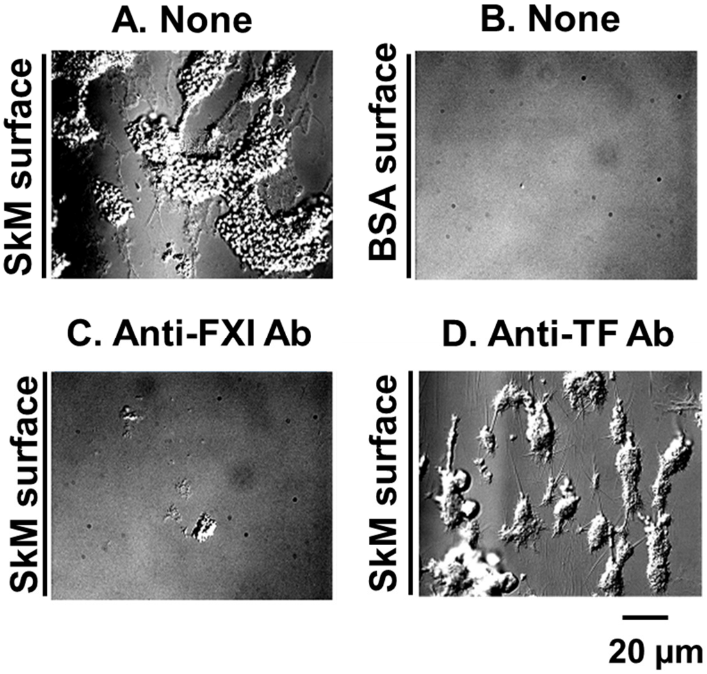

Figure S1. FXI is required for skeletal muscle myosin-induced blood clot formation in fresh flowing human blood. Skeletal muscle myosin (SkM)-coated glass capillary tubes (**A, C, D**) or bovine serum albumin (BSA)-coated (**B**) glass capillary tubes were exposed to flowing recalcified fresh human whole blood that contained additions of (**A, B**) no coagulation inhibitor ("None"); (**C**) anti-FXI mAb 1A6 (18); or (**D**) anti-TF mAb RbTF7 3A5 (1). Staining detects thrombi containing fibrin and aggregated platelets.
